# Association between Psychological Factors and Evacuation Status and the Incidence of Cardiovascular Diseases after the Great East Japan Earthquake: A Prospective Study of the Fukushima Health Management Survey

**DOI:** 10.3390/ijerph17217832

**Published:** 2020-10-26

**Authors:** Toshiki Sanoh, Eri Eguchi, Tetsuya Ohira, Fumikazu Hayashi, Masaharu Maeda, Seiji Yasumura, Yuriko Suzuki, Hirooki Yabe, Atsushi Takahashi, Kanae Takase, Mayumi Harigane, Takashi Hisamatsu, Keiki Ogino, Hideyuki Kanda, Kenji Kamiya

**Affiliations:** 1 Department of Public Health, Okayama University Graduate School of Medicine, Dentistry and Pharmaceutical Sciences, Okayama 700-8558, Japan; t.sanou@kousei-hp.or.jp (T.S.); hisamatsu@okayama-u.ac.jp (T.H.); kogino@md.okayama-u.ac.jp (K.O.); hkanda@okayama-u.ac.jp (H.K.); 2 Department of Epidemiology, Fukushima Medical University School of Medicine, Fukushima 960-1295, Japan; teoohira@fmu.ac.jp (T.O.); fhayashi@fmu.ac.jp (F.H.); 3 Radiation Medical Science Center for the Fukushima Health Management Survey, Fukushima Medical University, Fukushima 960-1295, Japan; masagen@fmu.ac.jp (M.M.); yasumura@fmu.ac.jp (S.Y.); hyabe@fmu.ac.jp (H.Y.); junior@fmu.ac.jp (A.T.); takase@fmu.ac.jp (K.T.); harigane@fmu.ac.jp (M.H.); kkamiya@fmu.ac.jp (K.K.); 4 Department of Disaster Psychiatry, Fukushima Medical University School of Medicine, Fukushima 960-1295, Japan; 5 Department of Public Health, Fukushima Medical University School of Medicine, Fukushima 960-1295, Japan; 6 National Institute of Mental Health, National Center of Neurology and Psychiatry, Tokyos 187-0031, Japan; yuriko-suzuki@amed.go.jp; 7 Department of Neuropsychiatry, Fukushima Medical University School of Medicine, Fukushima 960-1295, Japan; 8 Department of Gastroenterology, Fukushima Medical University School of Medicine, Fukushima 960-1295, Japan; 9 Department of Community Health and Public Health Nursing, Fukushima Medical University School of Nursing, Fukushima 960-1295, Japan; 10 Department of Environmental Medicine, Cooperative Medicine Unit, Research and Education Faculty, Medicine Science Cluster, Kochi Medical School Kochi University, Kochi 783-8505, Japan; 11 Research Institute for Radiation Biology and Medicine, Hiroshima University, Hiroshima 734-8553, Japan

**Keywords:** Great East Japan Earthquake, disaster, cardiovascular disease, psychological factors, evacuation, prospective study

## Abstract

Evidence regarding the effect of psychological factors and evacuation on cardiovascular disease occurrence after large-scale disasters is limited. This prospective study followed up a total of 37,810 Japanese men and women aged 30–89 years from the Fukushima Prefecture with no history of stroke or heart disease at baseline (2012), until 2017. This period included 3000 cardiovascular events recorded through questionnaires and death certificates. The participants’ psychological distress, trauma reaction, and evacuation status were defined, and divided into four groups based on combinations of psychological factors and evacuation status. We calculated the hazard ratios and 95% confidence intervals for only psychological, only evacuation, or both of them compared with neither using Cox proportional hazard models. Psychological factors along with evacuation resulted in approximately 5% to 25% higher magnitude of stroke and heart disease risk than psychological factors only among men. Compared to neither, the multivariable hazard ratios of those with both psychological distress and evacuation were 1.75 for stroke and 1.49 for heart disease, and those of both trauma reaction and evacuation were 2.01 and 1.57, respectively, among men. Evacuation combined with psychological factors increased the risk of stroke and heart disease risks especially in men after the Great East Japan Earthquake.

## 1. Introduction

The Great East Japan Earthquake on 11 March 2011 registered 9.0 on the Richter scale and caused a tsunami, resulting in a nuclear disaster in Fukushima Prefecture. Consequently, the prefectural government established evacuation zones in Fukushima, and more than 160,000 residents were forced to evacuate. As of 14 January 2020, more than 48,000 residents are still evacuated and bear psychological burdens and concerns regarding the lingering radiation risks. The local government therefore launched the Fukushima Health Management Survey (FHMS) to investigate and monitor the evacuees’ health condition [1]. To date, numerous studies have examined the relationship between disasters and cardiovascular disease (CVD) in individuals who experience such events [2,3,4,5,6]. Earlier studies on the Great East Japan Earthquake have revealed that the incidence of CVD, including out-of-hospital cardiac arrest, tachyarrhythmias, heart failure, acute coronary syndrome, and stroke, significantly increased following the disaster [7,8,9].

There is a complex association between disaster occurrence and CVD risk, and the available research indicates numerous risk factors, such as lifestyle-related, social, and psychological factors [10,11,12]. A number of studies have reported that the disaster was followed by cases of serious post-traumatic stress disorder (PTSD), which in turn induced CVD in certain individuals affected by the disaster [13,14]. According to evidence from a few studies, various modifiable risk factors for CVD in addition to psychological factors have been identified, one of which is evacuation status [15,16,17]. After a disaster, the resulting traumatic events and evacuation status affect individuals’ lifestyle and psychological state [18,19]. Earlier studies on the Great East Japan Earthquake have shown that evacuation status increases the incidence of hypertension, obesity, metabolic syndrome, diabetes, hyperlipidemia, liver dysfunction, chronic kidney disease and polycythemia, all of which are CVD risk factors [20,21,22,23,24,25,26,27]. Although a number of studies have focused on the relationship between psychological factors and CVD risk or between evacuation status and CVD risk, they have not clarified how evacuation status affects the association between psychological factors and CVD occurrence.

We therefore hypothesized that, since the occurrence of the Great East Japan Earthquake, psychological factors have become risk factors for CVD and that evacuation status might increase the effect of these factors. To our knowledge, no previous prospective study has examined the combined effect of psychological factors and evacuation status on CVD after the occurrence of large-scale disasters among worldwide populations. This large cohort study therefore prospectively examined the combined effects of psychological factors and evacuation status on CVD occurrence among male and female Japanese residents who experienced the earthquake in the Fukushima prefecture.

## 2. Materials and Methods

### 2.1. Participants

**Figure 1:**
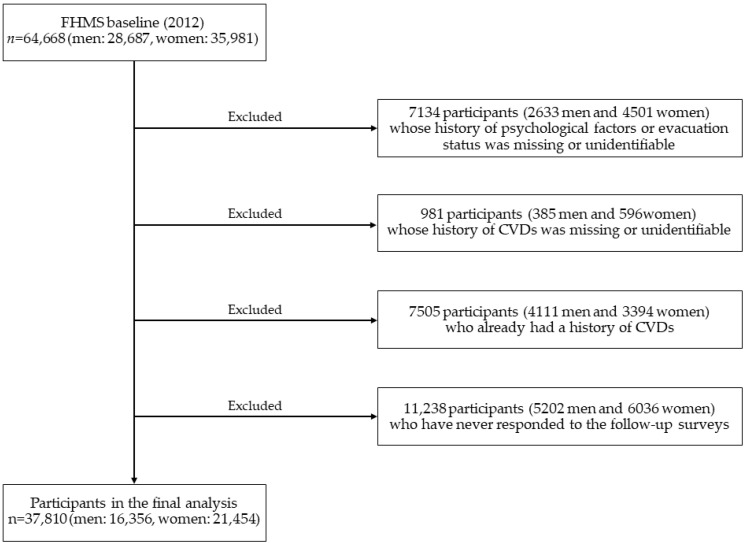
Flowchart of participant selection for this study of association between psychological factors and evacuation status and the incidence of cardiovascular diseases after the Great East Japan Earthquake.

To monitor the health status of Fukushima evacuees after the earthquake and to provide them with appropriate care, the Fukushima Medical University has been conducting an annual self-administered survey titled the “Mental Health and Lifestyle Survey [1].” One of its baseline surveys was conducted in January 2012 to investigate CVD onset, psychological factors, evacuation status, and lifestyle-related factors. Figure 1 provides a flow chart of the participant selection for the present study. In the survey, 64,668 Japanese individuals (28,687 men, 35,981 women) aged 30–89 years participated from 13 municipalities in the Fukushima Prefecture. Follow-up surveys assessing CVD onset were subsequently conducted once a year from baseline, and questionnaires were mailed to the participants annually. The present study excluded 2633 men and 4501 women whose history of psychological factors or evacuation status in 2012 was missing or unidentifiable, as well as 385 men and 596 women whose CVDs history in 2012 was missing or unidentifiable. The study also excluded 4111 men and 3394 women who already had a history of CVDs in 2012. The study further excluded 5202 men and 6036 women who never responded to the follow-up surveys. The final analysis therefore included 37,810 participants (16,356 men, 21,454 women).

The study was conducted based on the provisions of the Declaration of Helsinki, and the study protocol was approved by the Ethics Review Committees of Okayama University (No. 1803-022) and Fukushima Medical University (No. 1316, No. 2148, No. 2020-047). Submission of the self-administered questionnaires was considered as consent given by the participants for participating in the study.

### 2.2. Measurements

#### 2.2.1. Onset and Mortality of Cardiovascular Diseases

The study considered both onset and death as cardiovascular incidents. To measure the onset of CVDs (stroke or heart disease), the participants were presented once a year with the question, “Have you ever been diagnosed by a doctor with the following diseases?” and were asked to circle either “yes” or “no” for any applicable diseases [15,28,29,30]. The mortality data were forwarded to the public health departments of the respective areas before being centralized at the Ministry of Health, Labour and Welfare, and the underlying causes of death were coded based on the 10th revision of the International Classification for Diseases (ICD-10). The primary endpoints for the current analysis were death from stroke (ICD-10 codes I60–I69), heart disease (ICD-10 codes I20–I25 and I30–I52), and total CVD (stroke and heart disease).

#### 2.2.2. Psychological Factors and Evacuation Status

To assess the participants’ mental health status, we used the six-item Kessler Psychological Distress Scale (K6) and the Post-Traumatic Stress Disorder (PTSD) Checklist—Stressor-Specific Version (PCL-S). In particular, the K6 was employed to measure psychological distress and screen individuals for nonspecific serious mental illness [31]. The scale included questions on whether the participants had experienced any of the following six symptoms during the past 30 days: “feeling so sad that nothing could cheer you up,” “feeling nervous,” “feeling hopeless,” “feeling restless or fidgety,” “feeling everything was an effort” and “feeling worthless.” Each question was scored on a five-point Likert-type scale, with values ranging from 0 to 4. Scores ranged from 0 to 24, and higher scores indicated lower mental health status. The study validated and employed the Japanese version of K6 [32,33]. We defined psychological distress as scores ≥13.

The PCL-S was employed to assess the reaction to trauma to identify the participants’ current trauma-related symptoms [34]. The scale is a 17-item self-administered measure that detects PTSD, where each item is scored from 1 to 5 according to the responses “not at all,” “a little bit,” “moderately,” “quite a bit,” or “extremely,” respectively. Total scores range from 17 to 85, with higher scores indicating greater reaction to trauma. The study validated and employed the Japanese version of PCL-S [29,30]. We classified the participants with PCL-S scores ≥44 as having trauma reaction.

Evacuation status was assessed based on the living situations category. Participants were asked to select an answer from six options on their current living situations: “evacuation shelter,” “temporary housing,” “rental housing or apartment,” “a relative’s home,” “their own home,” or “other.” We defined those participants as evacuees who were currently living in or had lived in either an “evacuation shelter” or “temporary housing.”

#### 2.2.3. Lifestyle Behaviors, Social Factors, and Other Risk Factors for Cardiovascular Diseases

Lifestyle behaviors and social factors were employed as adjusted variables for the association between psychological factors or evacuation status and cardiovascular incidents. Lifestyle behaviors included the participants’ smoking status, alcohol consumption, physical activity and sleep quality. We assessed the participants’ smoking status using the question “Do you smoke (anything other than cigars and pipes)?” with the following options: “non-smoker,” “ex-smoker” and “current smoker.” Those who selected “current smoker” were considered current smokers. For the alcohol consumption category, individuals were asked, “Do you consume alcohol?” and the options were “less than once per month,” “ex-drinker” and “once or more per month.” Those who selected “once or more per month” were considered to have an alcohol consumption of once or more per month. The participants’ physical activity level was assessed by the question “Do you exercise regularly?” with the following options: “*≥*daily,” “2–4 times/week,” “weekly” and “almost never.” Those who selected “weekly” were considered to have a physical activity frequency of once or more a week. For the sleep quality category, the question was “Are you satisfied with the quality of sleep for the past month (regardless of the length of sleep)?”, and the options were “satisfied,” “slightly dissatisfied,” “very dissatisfied,” and “extremely dissatisfied.” Those who selected “slightly dissatisfied,” “very dissatisfied,” or “extremely dissatisfied” were considered unsatisfied with sleep.

The study considered job loss, loss of family members and a high perception of radiation risks to be social factors. The “job loss” and “loss of family members” categories included the following “yes” or “no” questions: “Did you become unemployed?” and “Did you lose loved ones in this earthquake?” A “yes” response to these questions was considered to be confirmation of job loss and loss of family members. Furthermore, for the “high perception of radiation risks” category, participants answered the following multiple-choice questions: “How likely do you think acute health problems (e.g., death within a month) will occur from the current radiation exposure?,” “How likely do you think health problems (e.g., cancer) will occur in the coming years due to the current radiation exposure?” and “To what extent do you think the current radiation exposure will affect future generations (children, grandchildren, etc.)?” The participants who answered “3” or “4” (where 1 corresponded to a low likelihood and 4 to a high likelihood) to any of these questions were considered to have a high perception of radiation risks.

The history of hypertension, hyperlipidemia, diabetes, and family history of CVD were measured. These CVD risk factors included the following “yes” or “no” questions: “Has your father or mother ever been diagnosed by a doctor with the following diseases?” A “yes” response to these questions was considered confirmation of these factors.

### 2.3. Statistical Analysis

The age-adjusted mean and prevalence of baseline variables of interest were compared between participants according to evacuation status, using analysis of covariance and logistic regression models [35]. Furthermore, *p* for differences were calculated between evacuees and non-evacuees. The number of cohorts was sufficient for the current analysis, and the risk of stroke was more significant among men; hence, the analyses in this study were stratified by gender.

The person-years of follow-up were calculated from the date of the response to the baseline questionnaire to the attainment of one of the following three possible endpoints: (1) a CVD event incidence (including death); (2) relocation from the study area; or (3) date of last response to the self-administered survey.

The participants were divided into four groups: participants with (1) neither evacuation status nor psychological factors (neither), (2) only psychological factors (only psychological), (3) only evacuation status (only evacuation) and (4) those with both psychological factors and evacuation status (both). The hazard ratios (HRs) and 95% confidence intervals (CIs) of cardiovascular incidence for the groups of only psychological, only evacuation, or both compared to neither were estimated using Cox proportional hazards models according to gender or evacuation status; subsequently, the relationship between each risk factors such as psychological distress or trauma reaction, and CVDs was examined. Similarly, the relationship between evacuation status and CVDs was evaluated. Furthermore, we investigate in detail the impact of gender or evacuation status-specific effect modifications on the association between psychological factors, such as psychological distress or trauma reaction, and CVDs. The *p*-values for interactions by gender or evacuation status were tested using the cross-product terms of gender and psychological distress or trauma reaction, or evacuation status and psychological distress or trauma reaction.

The adjustment variables included, age (continuous) for model 1, smoking status (dichotomous), alcohol consumption (dichotomous), physical activity (dichotomous), sleep quality (dichotomous), and job loss (dichotomous) in addition to model 1 for model 2, and history of hypertension (dichotomous), history of hyperlipidemia (dichotomous), history of diabetes (dichotomous) and family history of CVD (dichotomous) for model 3 in addition to model 2. We used SAS Version 9.4 (SAS Institute, Inc., Cary, NC, USA) for the statistical analysis. In this study, all statistical tests were two-tailed, and values of *p* < 0.05 were considered significant.

## 3. Results

During the 3.7-year mean follow-up period, the onset of all CVDs was reported for 2829 (1511 men and 1318 women), including 626 stroke onset cases (361 men and 265 women); 2203 heart disease onset cases (1150 men and 1053 women); and the death of 171 participants from all CVDs (86 men and 85 women), including 72 stroke (26 men and 46 women) and 99 heart disease (60 men and 39 women) death cases.

### 3.1. Cardiovascular Risk Factors of Participants at Baseline

Table 1 depicts the age-adjusted mean or prevalence of cardiovascular risk factors for the participants at baseline according to their evacuation status. Compared to non-evacuees, the average age of evacuees was lower, and evacuees had a higher prevalence of psychological distress, trauma reaction, current smoker, unsatisfied with sleep, job loss, loss of family members, high perception of radiation risks, history of hypertension and hyperlipidemia both in men and women.

### 3.2. Psychological Factors or Evacuation Status on Cardiovascular Diseases

Table 2 depicts the psychological factors or evacuation status-specific age-adjusted and multivariable HRs (95% CIs) of CVDs according to gender. For psychological distress, both men and women had an increased risk of stroke and heart disease. The respective HRs (95% CIs) of model 2 were 1.53 (1.22–1.92) and 1.40 (1.22–1.60) for men and 1.39 (1.12–1.73) and 1.40 (1.24–1.57) for women. Similarly, for trauma reaction, both men and women showed an increased risk of stroke and heart disease. Respective HRs of model 2 were 1.78 (1.49–2.14) and 1.43 (1.28–1.59) for men and 1.41 (1.17–1.70) and 1.35 (1.22–1.50) for women. For evacuation status, men showed significant or borderline significant increased risks for stroke and heart disease. The respective HRs of model 2 were 1.17 (0.98–1.40) and 1.11 (1.00–1.23). Women showed no increase in risk of stroke or heart disease based on their evacuation status. After further adjustments for other risk factors of CVDs: history of hypertension, history of hyperlipidemia, history of diabetes and family history of CVD, the HRs were up to 0.1 smaller, but no significant differences in overall results were observed. There were statistically significant interactions based on gender between evacuation status and stroke (*p* = 0.04).

### 3.3. Combination of Psychological Factors and Evacuation Status on Cardiovascular Diseases

Table 3 depicts the gender-specific age-adjusted and multivariable HRs (95% CIs) of CVDs according to the combination of psychological factors and evacuation status. Compared to those with neither psychological distress nor evacuation status, those with both had increased risks of stroke and heart disease and the increased magnitude due to evacuation was approximately 0.21 for stroke and 0.05 for heart disease in men. The respective HRs (95% CIs) of model 2 were 1.75 (1.26–2.44) for stroke and 1.49 (1.22–1.82) for heart disease. Compared to those with neither trauma reaction nor evacuation status, those with both had increased risks of stroke and heart disease, and the increased magnitude due to evacuation was approximately 0.25 for stroke and approximately 0.19 for heart disease in men. The respective HRs of model 2 were 2.01 (1.54–2.61) for stroke and 1.57 (1.34–1.84) for heart disease. Evacuation status in addition to psychological factors did not lead to an additional impact on CVDs in women. After the further adjustments, the HRs were up to 0.16 smaller, but no significant differences in overall results were observed.

Supplementary Table S1 depicts the gender-specific age-adjusted and multivariable HRs (95% CIs) of CVDs according to evacuation status. Men with psychological distress showed an increased risk of both stroke and heart disease, irrespective of evacuation status. The respective HRs (95% CIs) of model 2 were 1.49 (1.07–2.08) for evacuees and 1.54 (1.13–2.11) for non-evacuees for stroke, and 1.39 (1.14–1.69) vs. 1.41 (1.18–1.70) for heart disease. Similarly, men with trauma reaction showed an increased risk, and respective HRs of 1.79 (1.36–2.35) vs. 1.75 (1.37–2.24) for stroke, and 1.52 (1.29–1.79) vs. 1.35 (1.16–1.57) for heart disease. Women with psychological distress showed a significant or borderline significant increased risk of both stroke and heart disease, irrespective of evacuation status. The respective HRs (95% CIs) of model 2 were 1.50 (1.07–2.11) for evacuees and 1.32 (1.00–1.74) for non-evacuees for stroke, and 1.30 (1.09–1.56) vs. 1.47 (1.26–1.71) for heart disease. Women with a trauma reaction showed an increased risk of stroke for non-evacuees and heart disease irrespective of evacuation status. The respective HRs (95% CIs) were 1.53 (1.21–1.95) for non-evacuees for stroke, and 1.22 (1.04–1.43) vs. 1.46 (1.27–1.67) for heart disease. After the further adjustments, the HRs were up to 0.12 smaller, but no significant differences in overall results were observed.

## 4. Discussion

Using data from a large-scale prospective study among Japanese men and women aged 30–89 years in Fukushima after the Great East Japan Earthquake, we observed an association between psychological factors and evacuation status, and CVDs risk among individuals who experienced the disaster. Psychological factors along with evacuation status resulted in approximately 5% to 25% higher magnitude of stroke and heart disease risk in men than psychological factors alone.

The increased CVDs risks caused by psychological factors found in our study is consistent with the findings of earlier prospective cohort studies on individuals’ experiences following traumatic events such as disasters [12,13,14]. After the 2001 World Trade Center Disaster, a prospective cohort study was conducted in New York State, in which 46,346 participants were followed up for a mean period of 6.5 years per person. The adjustment HRs of patients with PTSD were approximately 1.5 times higher in men for cerebrovascular disease and 1.3 times higher in women for heart disease than for their counterparts without PTSD [36]. There have been similar reports on the relationship between psychological factors and CVDs following earthquake disasters as well. In a prospective cohort study conducted in China following the 2008 Sichuan earthquake, 404 participants were followed up for 2.0 years. This study found that the odds of participants having depressive symptoms caused by earthquake-related loss was approximately 1.6 times higher than in those who did not experience earthquake-related loss [37]. Furthermore, several studies have examined evacuation status following traumatic events and reported similar relationships [15,16,17]. In the FHMS, 73,433 people residing in the disaster zone of the Great East Japan Earthquake were analyzed using a cross-sectional design, and the odds ratios for cardiovascular symptoms, such as headache, dizziness, and shortness of breath, were found to be higher for evacuees than for non-evacuees [15]. However, these studies did not consider the combined impact of psychological factors and evacuation status on CVD occurrence.

This study identified a higher risk of CVDs occurrence among participants having both psychological factors and evacuation status compared with those having neither. Earlier research has demonstrated that disaster-induced psychological burdens and evacuation status are CVD risk factors [12,13,14,15,16,17]. Earlier studies also identified the loss of social networks as one of the risk factors for CVD [38,39]. In the Health Professionals Follow-up Study, which followed 51,529 American men for 10 years, the risk ratio for coronary heart disease was found to be approximately 1.8 times higher for socially isolated men than for men with the highest level of social networks [40]. Hence, the loss of social networks caused by changes in neighbors and a decrease in the frequency of social interaction can lead to future CVD risks. Therefore, the increased CVDs risk among people residing in the disaster zone of the Great East Japan Earthquake in Fukushima could have been caused in part by the loss of social networks among participants who were evacuated.

Furthermore, the increased risk of CVDs due to evacuation status added to psychological factors was observed in men but not in women. To date, some studies after the Great East Japan Earthquake have focused on the association between evacuation status and risk factors for CVD analyzed by gender. In the FHMS, a prospective cohort study from 2011 to 2013 showed an association between evacuation status and hypertension and found the age-adjusted HRs in men to be approximately 1.2 times higher than those in women [20]. The same association was observed for overweight, metabolic syndrome, and diabetes, particularly in men [21,22,23]. These diseases are major risk factors for CVD. Hence, these studies suggest that men might be more susceptible to CVD risk from evacuation status than women following disaster occurrence. Furthermore, symptoms such as hypertension, overweight, metabolic syndrome, or diabetes among male evacuees of the Great East Japan Earthquake might be associated with an increased risk of CVD. In fact, in this study, adjusting for history of hypertension, hyperlipidemia, diabetes, or family history of CVD resulted in slightly smaller HRs, indicating that these items were partly responsible for CVD development.

This study has the following salient features: (1) identified a large population-based cohort, including 38,710 participants in the affected area immediately after the occurrence of the Great East Japan Earthquake; (2) adopted a longitudinal design (2012–2017); (3) considered the combined impact of psychological factors and evacuation status on both men and women; and (4) adjusted for a range of potential confounders including lifestyle and work-related factors. However, the study also has some limitations. (1) To clarify CVD onset, the study used an annual self-administered survey, whose overall response rate was not very high (77.1%). However, it considered to have not significantly affect the study’s results. In fact, although the study excluded 5202 men and 6036 women who never responded to the follow-up surveys, there was no substantial difference in CVD risk factors at baseline between those who responded to follow-up and those who did not. (2) In this study, evacuation status was defined based on living situations, and evacuees were defined as those who currently live or have lived in “evacuation shelter” or “temporary housing.” Therefore, it does not include all the evacuation areas in Fukushima Prefecture designated by the government. It should also be noted that there is a large percentage of overlap (20.7% of males and 18.8% of females) between those who have lived in “evacuation shelter” and “temporary housing.” However, because it is based on actual evacuation life, the psychological physical, or social burden is likely to be more accurately reflected based on the living situations. (3) Body mass index (BMI) and waist circumference, both of which are risk factors for CVD, were not assessed in the baseline year (2012), and therefore could not be added as covariates. However, a sensitivity analysis using BMI data from this surveys in 2013 did not change the results.

## 5. Conclusions

In summary, this study combined evacuation status with psychological factors, which enabled us to identify a 5% to 25% increased risk of stroke and heart disease in men after the Great East Japan Earthquake in Fukushima. This study provides useful information for healthcare policymakers who plan CVD prevention strategies after major disasters and emphasizes the need for further monitoring of affected areas, particularly with respect to evacuees with psychological burdens. Future research should extend the follow-up period and consider long-term associations in greater detail.

## Figures and Tables

**Table 1:** Age-adjusted mean or prevalence of cardiovascular risk factors for the participants at baseline according to their evacuation status.

Cardiovascular Risk Factors	Evacuee	Non-Evacuee	*p* for Difference
**Men**			
Number at risk	5990	10,336	
Age (years)	57.6	58.4	<0.001
Psychological distress (%)	13.6	9.2	<0.001
Trauma reaction (%)	21.1	14.4	<0.001
Current smoker (%)	35.6	33.2	0.03
Alcohol consumption ≥ once/month (%)	68.6	71.5	<0.001
Physical activity ≥ once/month (%)	47.8	47.5	0.38
Unsatisfied with sleep (%)	64.9	56.5	<0.001
Job loss (%)	28.0	13.9	<0.001
Loss of family members (%)	23.6	16.9	<0.001
High perception of radiation risks (%)	61.4	56.4	<0.001
History of hypertension (%)	46.4	44.2	<0.001
History of hyperlipidemia (%)	39.7	37.0	<0.001
History of diabetes (%)	22.6	21.1	<0.001
Family history of CVD	35.2	35.8	0.47
**Women**			
Number at risk	8172	13,282	
Age (years)	55.7	56.5	<0.001
Psychological distress (%)	18.5	13.9	<0.001
Trauma reaction (%)	27.1	20.5	<0.001
Current smoker (%)	10.7	9.3	0.02
Alcohol consumption ≥ once/month (%)	30.6	30.6	0.38
Physical activity ≥ once/month (%)	52.3	53.3	0.002
Unsatisfied with sleep (%)	75.7	67.1	<0.001
Job loss (%)	33.9	18.8	<0.001
Loss of family members (%)	24.8	18.2	<0.001
High perception of radiation risks (%)	67.5	62.5	<0.001
History of hypertension (%)	37.6	36.9	<0.001
History of hyperlipidemia (%)	35.7	34.7	0.002
History of diabetes (%)	14.7	15.1	0.67
Family history of CVD	37.2	38.2	0.14

Abbreviation: CVD, cardiovascular disease.

**Table 2:** Psychological factors or evacuation status-specific age-adjusted and multivariable HRs (95% CIs) of CVDs according to gender.

	Men	Women	*p* for Interaction ^a^
Person-years	59,087	79,350	
**Psychological distress**			
Total CVD (*n*)	301	449	
Incidence rate/1000 person-years	5.09	5.66	
Model 1 ^b^	1.48 (1.31–1.67)	1.48 (1.33–1.64)	0.93
Model 2 ^c^	1.34 (1.18–1.52)	1.39 (1.25–1.55)	0.90
Model 3 ^d^	1.30 (1.15–1.48)	1.36 (1.22–1.51)	0.94
Stroke (n)	92	111	
Incidence rate/1000 person-years	1.56	1.40	
Model 1	1.66 (1.33–2.07)	1.48 (1.20–1.83)	0.45
Model 2	1.53 (1.22–1.92)	1.39 (1.12–1.73)	0.49
Model 3	1.47 (1.17–1.85)	1.34 (1.08–1.67)	0.45
Heart disease (n)	262	381	
Incidence rate/1000 person-years	4.43	4.80	
Model 1	1.56 (1.37–1.77)	1.49 (1.33–1.67)	0.59
Model 2	1.40 (1.22–1.60)	1.40 (1.24–1.57)	0.73
Model 3	1.36 (1.19–1.56)	1.36 (1.21–1.53)	0.70
**Trauma reaction**			
Total CVD (*n*)	523	662	
Incidence rate/1000 person-years	8.85	8.34	
Model 1	1.54 (1.40–1.70)	1.43 (1.30–1.57)	0.37
Model 2	1.42 (1.29–1.52)	1.35 (1.23–1.49)	0.41
Model 3	1.37 (1.23–1.51)	1.31 (1.19–1.44)	0.37
Stroke (n)	174	170	
Incidence rate/1000 person-years	2.94	2.14	
Model 1	1.86 (1.56–2.22)	1.49 (1.24–1.79)	0.09
Model 2	1.78 (1.49–2.14)	1.41 (1.17–1.70)	0.08
Model 3	1.68 (1.40–2.02)	1.35 (1.12–1.64)	0.07
Heart disease (n)	437	559	
Incidence rate/1000 person-years	7.40	7.04	
Model 1	1.56 (1.40–1.73)	1.44 (1.30–1.59)	0.39
Model 2	1.43 (1.28–1.59)	1.35 (1.22–1.50)	0.45
Model 3	1.37 (1.23–1.53)	1.31 (1.18–1.46)	0.41
**Evacuation status**			
Total CVD (*n*)	871	819	
Incidence rate/1000 person-years	14.74	10.32	
Model 1	1.18 (1.09–1.29)	1.07 (0.98–1.17)	0.10
Model 2	1.13 (1.02–1.24)	1.03 (0.94–1.14)	0.13
Model 3	1.11 (1.01–1.22)	1.03 (0.94–1.14)	0.19
Stroke (n)	247	182	
Incidence rate/1000 person-years	4.18	2.29	
Model 1	1.21 (1.03–1.42)	0.93 (0.77–1.11)	0.03
Model 2	1.17 (0.98–1.40)	0.90 (0.74–1.10)	0.04
Model 3	1.15 (0.97–1.38)	0.90 (0.74–1.10)	0.06
Heart disease (n)	719	698	
Incidence rate/1000 person-years	12.17	8.80	
Model 1	1.17 (1.06–1.28)	1.10 (1.00–1.21)	0.29
Model 2	1.11 (1.00–1.23)	1.04 (0.93–1.16)	0.35
Model 3	1.09 (0.98–1.21)	1.04 (0.93–1.16)	0.43

Abbreviations: CVD, cardiovascular disease; HR, hazards ratio; CI, confidence interval. Notes: values in parentheses indicate 95% confidence intervals. ^a^ *p* for interaction was calculated for the cross-product terms of gender and psychological distress, trauma reaction or evacuation status on CVDs. ^b^ Adjusted for age. ^c^ Adjusted for smoking status, alcohol consumption, physical activity, sleep quality and job loss in addition to Model 1. ^d^ Adjusted for history of hypertension, history of hyperlipidemia, history of diabetes and family history of CVD in addition to Model 2.

**Table 3:** Gender-specific age-adjusted and multivariable HRs (95% CIs) of CVDs according to the combination of psychological factors and evacuation status.

	Neither	Only Psychological	Only Evacuation	Both
**Men**				
Person-years	31,101	6218	16,702	5067
**Psychological distress**				
Total CVD (*n*)	1162	156	726	145
Model 1 ^a^	1.00	1.50 (1.27–1.77)	1.17 (1.07–1.29)	1.65 (1.39–1.96)
Model 2 ^b^	1.00	1.37 (1.15–1.62)	1.13 (1.02–1.25)	1.46 (1.22–1.76)
Model 3 ^c^	1.00	1.34 (1.13–1.59)	1.12 (1.01–1.24)	1.39 (1.16–1.67)
Stroke (n)	328	47	202	45
Model 1	1.00	1.65 (1.22–2.25)	1.18 (0.99–1.41)	1.89 (1.38–2.58)
Model 2	1.00	1.54 (1.13–2.10)	1.17 (0.96–1.41)	1.75 (1.26–2.44)
Model 3	1.00	1.50 (1.10–2.04)	1.15 (0.95–1.39)	1.64 (1.18–2.29)
Heart disease (n)	960	138	595	124
Model 1	1.00	1.60 (1.34–1.91)	1.16 (1.05–1.28)	1.70 (1.41–2.05)
Model 2	1.00	1.44 (1.21–1.73)	1.11 (0.99–1.24)	1.49 (1.22–1.82)
Model 3	1.00	1.42 (1.19–1.70)	1.10 (0.98–1.23)	1.42 (1.17–1.74)
**Trauma reaction**				
Total CVD (*n*)	1057	261	609	262
Model 1	1.00	1.48 (1.30–1.71)	1.13 (1.02–1.25)	1.75 (1.53–2.00)
Model 2	1.00	1.38 (1.21–1.59)	1.09 (0.98–1.22)	1.58 (1.37–1.84)
Model 3	1.00	1.34 (1.16–1.53)	1.08 (0.97–1.21)	1.50 (1.29–1.74)
Stroke (n)	287	88	161	86
Model 1	1.00	1.83 (1.44–2.32)	1.14 (0.94–1.38)	2.08 (1.64–2.65)
Model 2	1.00	1.76 (1.38–2.24)	1.13 (0.92–1.39)	2.01 (1.54–2.61)
Model 3	1.00	1.69 (1.32–2.15)	1.12 (0.91–1.38)	1.85 (1.42–2.40)
Heart disease (n)	880	218	500	219
Model 1	1.00	1.49 (1.28–1.73)	1.11 (0.99–1.24)	1.76 (1.52–2.04)
Model 2	1.00	1.38 (1.19–1.60)	1.07 (0.95–1.20)	1.57 (1.34–1.84)
Model 3	1.00	1.33 (1.14–1.55)	1.06 (0.94–1.19)	1.49 (1.27–1.75)
**Women**				
Person-years	37,141	11,498	21,371	9339
**Psychological distress**				
Total CVD (*n*)	1002	257	627	192
Model 1	1.00	1.53 (1.34–1.76)	1.08 (0.98–1.19)	1.50 (1.29–1.75)
Model 2	1.00	1.45 (1.26–1.66)	1.05 (0.94–1.17)	1.38 (1.18–1.63)
Model 3	1.00	1.41 (1.22–1.62)	1.04 (0.94–1.16)	1.35 (1.15–1.59)
Stroke (n)	265	64	135	47
Model 1	1.00	1.44 (1.09–1.89)	0.89 (0.73–1.10)	1.41 (1.03–1.92)
Model 2	1.00	1.34 (1.01–1.77)	0.87 (0.70–1.09)	1.29 (0.93–1.79)
Model 3	1.00	1.28 (0.97–1.69)	0.87 (0.69–1.08)	1.26 (0.91–1.75)
Heart disease (n)	832	218	522	163
Model 1	1.00	1.56 (1.35–1.82)	1.11 (0.99–1.23)	1.53 (1.29–1.81)
Model 2	1.00	1.47 (1.26–1.71)	1.06 (0.94–1.19)	1.38 (1.15–1.65)
Model 3	1.00	1.43 (1.22–1.66)	1.06 (0.94–1.19)	1.35 (1.13–1.61)
**Trauma reaction**				
Total CVD (*n*)	880	379	536	283
Model 1	1.00	1.55 (1.37–1.75)	1.11 (1.00–1.24)	1.41 (1.23–1.61)
Model 2	1.00	1.47 (1.30–1.66)	1.08 (0.97–1.22)	1.30 (1.13–1.50)
Model 3	1.00	1.42 (1.26–1.61)	1.08 (0.97–1.22)	1.27 (1.10–1.46)
Stroke (n)	225	104	116	283
Model 1	1.00	1.63 (1.30–2.06)	0.97 (0.77–1.21)	1.27 (0.97–1.67)
Model 2	1.00	1.55 (1.22–1.96)	0.95 (0.75–1.20)	1.17 (0.88–1.57)
Model 3	1.00	1.48 (1.16–1.87)	0.95 (0.75–1.20)	1.14 (0.85–1.52)
Heart disease (n)	735	315	454	244
Model 1	1.00	1.54 (1.35–1.76)	1.13 (1.00–1.27)	1.46 (1.26–1.69)
Model 2	1.00	1.45 (1.27–1.66)	1.08 (0.95–1.23)	1.32 (1.13–1.54)
Model 3	1.00	1.41 (1.23–1.61)	1.08 (0.95–1.22)	1.28 (1.09–1.50)

Abbreviations: CVD, cardiovascular disease; HR, hazards ratio; CI, confidence interval. Note: ^a^ Adjusted for age. ^b^ Adjusted for smoking status, alcohol consumption, physical activity, sleep quality, and job loss in addition to Model 1. ^c^ Adjusted for history of hypertension, history of hyperlipidemia, history of diabetes and family history of CVD in addition to Model 2.

## Supplementary Materials

The following are available online at https://www.mdpi.com/1660-4601/17/21/7832/s1, Table S1: Gender-specific age-adjusted and multivariable HRs (95% CIs) of CVDs according to evacuation status.
