# Supplementary material for: Association between Psychological Factors and Evacuation Status and the Incidence of Cardiovascular Diseases after the Great East Japan Earthquake: A Prospective Study of the Fukushima Health Management Survey
Source: Int J Environ Res Public Health. 2020 Oct 26;17(21):7832. doi: 10.3390/ijerph17217832 (PMC7663529; doi:10.3390/ijerph17217832)
Supplement: Supplementary file 1 [file ijerph-17-07832-s001.pdf]

Table S1. Gender-specific age-adjusted and multivariable HRs (95% CIs) of CVDs according to evacuation status.

	Evacuee	Non-evacuee	p for interaction [a]
**Men**			
Person-years	21,768	37,319	
**Psychological distress**			
Total CVD (n)	145	156	
Model 1 [b]	1.41 (1.18–1.68)	1.50 (1.27–1.77)	0.61
Model 2 [c]	1.32 (1.10–1.59)	1.35 (1.14–1.60)	0.69
Model 3 [d]	1.27 (1.06–1.52)	1.33 (1.12–1.58)	0.56
Stroke (n)	45	47	
Model 1	1.59 (1.15–2.20)	1.65 (1.21–2.24)	0.88
Model 2	1.49 (1.07–2.08)	1.54 (1.13–2.11)	0.92
Model 3	1.40 (1.00–1.95)	1.50 (1.10–2.06)	0.82
Heart disease (n)	124	138	
Model 1	1.46 (1.21–1.77)	1.60 (1.34–1.91)	0.51
Model 2	1.39 (1.14–1.69)	1.41 (1.18–1.70)	0.59
Model 3	1.33 (1.09–1.63)	1.39 (1.16–1.67)	0.49
**Trauma reaction**			
Total CVD (n)	262	261	
Model 1	1.55 (1.34–1.79)	1.48 (1.29–1.70)	0.66
Model 2	1.48 (1.27–1.72)	1.37 (1.19–1.57)	0.65
Model 3	1.41 (1.21–1.64)	1.32 (1.15–1.52)	0.73
Stroke (n)	86	88	
Model 1	1.84 (1.42–2.40)	1.82 (1.44–2.32)	0.98
Model 2	1.79 (1.36–2.35)	1.75 (1.37–2.24)	0.95
Model 3	1.67 (1.27–2.19)	1.65 (1.29–2.11)	0.89
Heart disease (n)	163	218	
Model 1	1.58 (1.35–1.86)	1.49 (1.28–1.73)	0.57
Model 2	1.52 (1.29–1.79)	1.35 (1.16–1.57)	0.56
Model 3	1.45 (1.23–1.72)	1.31 (1.12–1.52)	0.61
**Women**			
Person-years	30,710	48,639	
**Psychological distress**			
Total CVD (n)	192	257	
Model 1 [b]	1.39 (1.18–1.62)	1.53 (1.34–1.76)	0.36
Model 2 [c]	1.33 (1.12–1.56)	1.44 (1.26–1.66)	0.41
Model 3 [d]	1.30 (1.10–1.53)	1.40 (1.22–1.61)	0.27
Stroke (n)	47	64	
Model 1	1.58 (1.13–2.20)	1.43 (1.08–1.87)	0.67
Model 2	1.50 (1.07–2.11)	1.32 (1.00–1.74)	0.63
Model 3	1.49 (1.06–1.52)	1.23 (0.93–1.64)	0.56
Heart disease (n)	143	203	
Model 1	1.38 (1.16–1.64)	1.57 (1.35–1.82)	0.29
Model 2	1.30 (1.09–1.56)	1.47 (1.26–1.71)	0.32
Model 3	1.27 (1.06–1.52)	1.43 (1.23–1.67)	0.34
**Trauma reaction**			

Total CVD (n)	283	379	
Model 1	1.27 (1.10–1.47)	1.54 (1.37–1.74)	0.04
Model 2	1.20 (1.04–1.40)	1.47 (1.30–1.66)	0.04
Model 3	1.17 (1.01–1.36)	1.42 (1.26–1.61)	0.04
Stroke (n)	66	104	
Model 1	1.35 (1.00–1.83)	1.62 (1.28–2.05)	0.27
Model 2	1.26 (0.92–1.73)	1.53 (1.21–1.95)	0.25
Model 3	1.23 (0.90–1.68)	1.44 (1.13–1.84)	0.29
Heart disease (n)	244	315	
Model 1	1.30 (1.11–1.52)	1.54 (1.35–1.76)	0.08
Model 2	1.22 (1.04–1.43)	1.46 (1.27–1.67)	0.09
Model 3	1.19 (1.01–1.40)	1.41 (1.23–1.62)	0.10

Abbreviations: CVD, cardiovascular disease; HR, hazards ratio; CI, confidence interval. Notes: values in parentheses indicate 95% confidence intervals. [a] *p* for interaction was calculated for the cross-product terms of gender and psychological distress, trauma reaction or evacuation status on CVDs. [b] Adjusted for age. [c] Adjusted for smoking status, alcohol consumption, physical activity, sleep quality and job loss in addition to Model 1. [d] Adjusted for history of hypertension, history of hyperlipidemia, history of diabetes and family history of CVD in addition to Model 2.
